# Training Load, Injuries, and Well-Being in Youth Padel Players: A Cross-Sectional Study

**DOI:** 10.3390/sports13100356

**Published:** 2025-10-07

**Authors:** Sofia Ryman Augustsson, Lisa Durdel

**Affiliations:** 1 Department of Sport Science, Linnaeus University, 392 31 Kalmar, Sweden; 2 Region Kalmar, 392 54 Kalmar, Sweden; lisa.durdel@regionkalmar.se

**Keywords:** injury prevalence, injury incidence, risk factors, youth athletes

## Abstract

The aim of this study was to explore the prevalence of acute and overuse injuries, as well as risk factors, training load and well-being, in male and female youth padel players. Using a cross-sectional design, data were collected from 104 players (aged 15–20) via a web-based form. Players reported injuries, exposure and rating of perceived exertion (RPE), demographics (age and sex), and perceived well-being. Overuse injury severity was scored per body region (0–25), yielding a total possible score of 125. A total of six acute and 49 overuse injuries were recorded, corresponding to a prevalence of 0.53 injuries per player during a one-week recall period. Most injuries affected the knee, while the foot and lower leg had the highest severity scores (median = 44). Female players reported slightly higher stress levels (median 3) than males (median 2: *p* = 0.01), though no other well-being or training load differences were found. Injured players had significantly higher total wellness scores, indicating worse well-being, compared to non-injured players (median 10 vs. 9, *p* = 0.03). In conclusion, overuse injuries, particularly to the knee, were most common. Higher perceived stress and poorer wellness scores may be linked to injury risk, underlining the importance of monitoring well-being in youth padel athletes.

## 1. Introduction

Padel has rapidly emerged as a popular sport worldwide and is likely contributing to increased physical activity while reducing sedentary behavior [1]. In Sweden, the sport’s growth has been substantial; by May 2021, padel was officially recognized by the Swedish Sports Confederation. As of 2024, the Swedish Padel Federation reported approximately 50,000 members, and at least 500,000 Swedes had played padel on one or more occasions [2]. Participation among adolescents, particularly those aged 14–18, has increased steadily, with approximately 800 licensed youth players currently registered in Sweden [2].

Beyond improving physical capacity, padel offers an engaging and enjoyable activity for children and adolescents, supporting the development of motor and cognitive skills and promoting overall health [1]. It is characterized as an intermittent sport involving rapid changes in direction, varying game pace, and frequent start-stop movements [3,4]. A limited number of studies have investigated the physical demands, playing characteristics, and capacities of youth padel players [5,6,7]. One study examining match activity in young players found that rallies tended to be longer, rest intervals more extended, and the number of strokes per rally higher compared to other racket sports, resulting in lower overall physical exertion [6]. Players under 16 years of age appear to experience lower physical demands than their older counterparts, and notable sex-based differences in play have also been observed [6]. For instance, girls tend to use the lob more frequently than boys [6], while women generally demonstrate a lower stroke frequency and slightly longer rally durations compared to men [3,4]. These age- and sex-based differences in physical demands and technical skills underscore the need for tailored training approaches to enhance performance and reduce injury risk [6].

Several studies have documented both overuse and traumatic (acute) injuries associated with padel [8,9,10]. Overuse injuries commonly affect the knee, elbow, foot, and back, with shoulder issues having the greatest impact on performance [9]. In addition, acute injuries such as ligament sprains and muscle strains are common [9]. However, data on injury patterns in youth padel players are scarce. In a study comparing injury profiles across age groups, Castillo-Lozano and Casuso-Holgado [11] found that senior players (aged 55–67) had a higher incidence of elbow and knee injuries, whereas junior players (aged 14–20) more frequently reported lower back pain. Thus, there appears to be a difference in the injury panorama between youth and adult players. However, to our knowledge, this is the only study that has investigated the injury panorama in youth padel players, highlighting a gap in the existing research.

The cause of sports injuries is multifactorial and several risk factors for padel injuries have been presented in the literature [8,9,10]. Suggested risk factors in padel include footwear [8] and racket characteristics [9]. Another proposed risk factor is age, particularly because many participants are middle-aged recreational players with limited prior training experience [12]. Still, young athletes are often exposed to high training volumes, intense workloads, and frequent competition participation in efforts to support long-term athletic development. However, such high levels of exposure can increase the risk of musculoskeletal injuries. Additionally, compromised well-being, resulting from factors like insufficient recovery, psychological stress, or poor sleep, may further contribute to the injury panorama in this population [13,14,15,16,17]. Lower well-being and higher training load have previously been identified as risk factors for injury in youth athletes [18]. One study reported that high training load during the previous week, measured using session rating of perceived exertion (sRPE) and acute/chronic workload ratio (ACWR), was associated with injury risk in the following week in junior tennis players [19]. This aligns with findings in other youth sports, where increased training loads and poor perceived well-being have been linked to an elevated injury risk [13,20]. For example, a rapid increase in training load has been associated with higher injury rates among youth athletes [13], while external stressors have been implicated in traumatic knee injuries in female youth athletes [21]. Despite the growing popularity of padel among young people, research on training load and well-being, as well as on injury patterns and other risk factors such as environmental influences and racket changes, remains limited in youth padel players.

Therefore, the aim of this study was to explore the prevalence of both acute and overuse injuries, as well as risk factors including training load and well-being, in male and female youth padel players.

## 2. Materials and Methods

### 2.1. Study Design and Participants

**Figure 1:**
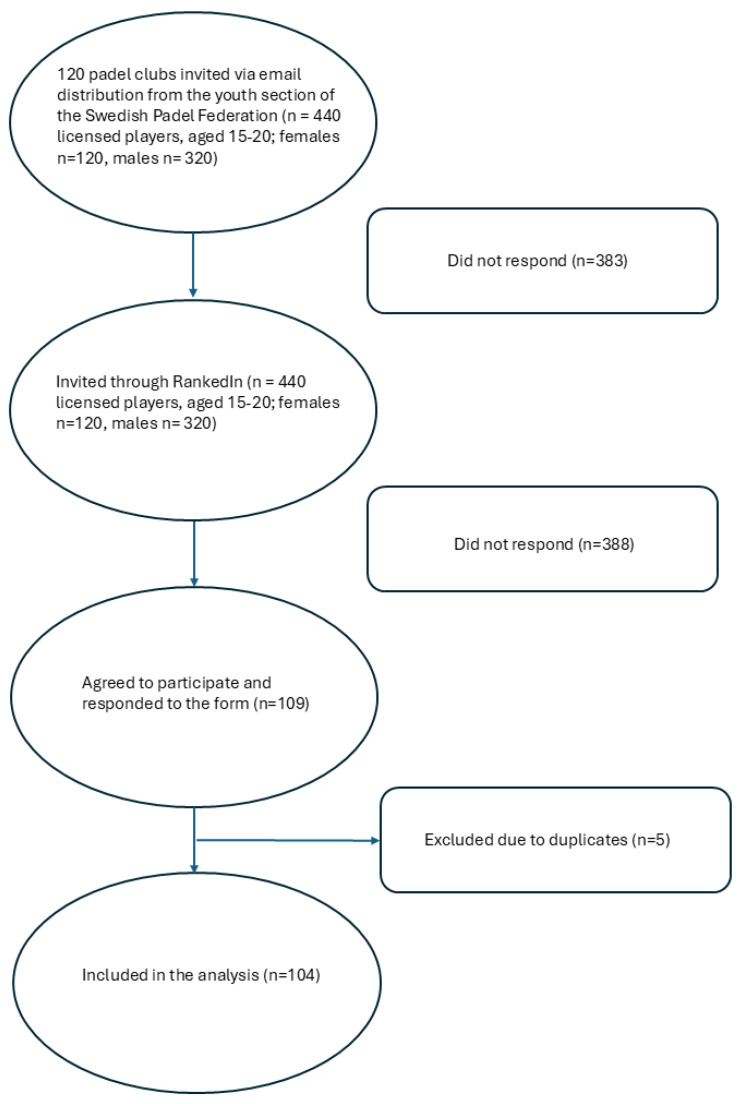
Flow chart of study participants.

Data for this cross-sectional study, conducted in accordance with the STROBE statement [22] were collected from January 2024 to April 2025 using a web-based form. Participants included male and female youth padel players aged 15–20 years who were members of a padel club in Sweden. Exclusion criteria were individuals younger than 15 or older than 20 years, as well as those unable to read or understand Swedish. Players were recruited via email through a contact person at the youth section of the Swedish Padel Association, who distributed the survey to all padel clubs in Sweden. Additional recruitment was conducted through RankedIn. Rankedin is a digital system and platform used to manage and organize padel tournaments, including registration, rankings, memberships, and payments. Rankedin is the Swedish Padel Federation’s official tournament tool to manage all sanctioned competitions and player licenses [23]. According to the Swedish Padel Association’s rankings, players in the Boys Youth 16 years (BU16), Girls Youth 16 years (GU16), Boys Youth 18 years (BU18), and Girls Youth 18 years (GU18) categories were contacted via direct messages on RankedIn, while players aged 18–20 were identified through the ‘men-main’ and ‘women-main’ categories. While players are assigned to different RankedIn categories based on both age and ranking level, this study considered only age as a criterion for inclusion, with ranking level having no influence. A total of 440 players (120 females) were invited through the platform (Figure 1). Reminder emails were sent to non-respondents one and two weeks after the initial invitation. The invitation included both a web link and a QR code directing participants to the form.

### 2.2. Procedure and Outcome Measures

Exposure and injuries were recorded using a previously developed padel-specific form, which combined registration protocols for both overuse injuries [24] and acute injuries [9] (Supplementary S1). Injuries were classified as either acute, defined as a sudden onset of pain following a specific, identifiable event, or as resulting from overuse. For overuse injuries, players assessed the severity of symptoms for each body region on a 0–100 scale, following a previously established protocol [9,25]. The form was piloted in advance to ensure feasibility, clarity, and relevance, thereby establishing face validity for the youth player cohort. The survey was distributed via SurveyMonkey^®^ and included an initial page providing study information and obtaining consent to participate. After providing consent, players were instructed to report any musculoskeletal complaints or injuries, including head or eye trauma, experienced during the past week. The form also collected demographic data (age and sex), exposure (match and training hours), and rating of perceived exertion (RPE), assessed using the Borg CR10 scale [26,27], during the past week. Additionally, perceived well-being was evaluated using a modified version of the Hooper Index questionnaire, a validated tool for monitoring psychometric status in athletes [28,29]. This instrument assesses perceived stress, sleep quality, fatigue, and muscle soreness on a seven-point Likert scale, with the total Hooper Score (HS) calculated as the sum of these four domains, giving a comprehensive index of the player’s overall psychophysiological state [28]. For the purposes of this study, only stress, sleep quality, and fatigue during the past week were recorded. For sleep, a score of 1 indicated extremely good sleep and 7 indicated extremely poor sleep. For stress, 1 represented no stress at all, while 7 denoted extreme stress. For fatigue, 1 corresponded to feeling very well-rested, and 7 to extreme fatigue. These ratings contributed to a total wellness score, with a maximum of 21 points. Finally, players were asked whether they had played indoors or outdoors, and whether they had changed their racket during the past week. To ensure anonymity, IP address tracking was disabled, and completed forms were transferred to the secure Swedish storage server Sunet Drive before deletion.

### 2.3. Statistical Analysis

Statistical analyses were conducted using IBM SPSS Statistics for Windows, Version 30.0 (IBM Corp., Armonk, NY, USA). Injury prevalence was defined as the number of injuries reported per player. Internal training load (ITL) was calculated by multiplying RPE by the duration of training and games in minutes, expressed as arbitrary units (AU) [19]. To assess whether continuous data (age, training/game hours, and ITL) were normally distributed, a Shapiro–Wilk test of normality was performed. The test indicated that data for both female and male players were not normally distributed (*p* < 0.001); therefore, non-parametric statistics were applied. Descriptive statistics are reported as means and standard deviations (SD) for continuous data to allow comparison with previous research, while medians with quartiles are presented for categorical variables and proportions (%) where appropriate. Group comparisons were performed using Mann–Whitney U test and Fisher’s exact test was used to compare the proportion of injured versus non-injured players (yes/no) by sex (female/male) and ‘indoors or outdoors’ (yes/no) as well as upper extremity injury (yes/no) by ‘changed racket’ (yes/no). Binary logistic regression was used to analyze the association between injury prevalence (yes = 1, no = 0) and potential associated factors, including training load, HS, and scores from individual well-being categories (stress, sleep, and fatigue). Odds ratios and their 95% confidence intervals were calculated. In addition, Cohen’s d was calculated to evaluate changes in the significant outcomes between groups, with effect sizes classified as small (d < 0.50), medium (0.50–0.80), and large (d > 0.80) [30].

This observational study is exploratory in nature, as limited prior data are available. Therefore, all licensed Swedish youth players were invited to participate, and no sample size calculation was performed. Statistical significance was set at *p* < 0.05.

## 3. Results

A total of 104 eligible youth padel players responded to the form, representing approximately 24% of the total population of Swedish licensed players aged 15–20. The total self-reported exposure time for training and matches was 1066 h, corresponding to an average of 10 h per player for one week (Table 1). A significant difference in age was observed between female and male players (*p* = 0.005). No significant differences were found between the sexes in terms of total exposure time, number of training hours, amount of match play, ITL or RPE (*p* ≥ 0.541).

### 3.1. Injury Panorama

A total of 35 players had experienced an injury during the past week and a self-reported injury prevalence of 0.53 injuries per player was observed. Among female players, 9 out of 36 sustained a total of 17 injuries, corresponding to a prevalence of 0.47 injuries per player. In comparison, male players reported 38 injuries among 26 individuals, resulting in a prevalence of 0.56 injuries per player. No significant differences were found between female and male players regarding the number of injured players or the total number of injuries (*p* ≥ 0.19). In total, six acute injuries were recorded: one eye injury, one shoulder ligament injury, one thigh muscle strain, one foot fracture, and two ankle sprains. Two of the athletes who sustained acute injuries were additionally affected by an overuse injury. Forty-nine overuse injuries were reported, with the majority affecting the knee. However, the foot and lower leg were associated with the highest overall severity score (median = 44) (Table 2).

### 3.2. Risk Factors and Well-Being

Six players reported having played outdoors, while another six had played both outdoors and indoors. Additionally, six players had changed their racket during the past week. No significant association was found between playing outdoors and injury risk (*p* = 0.98), nor between changing racket and the risk of upper extremity injury (*p* = 0.19). Injured players reported a slightly higher total wellness score (median 10, Q1–3 3), indicating poorer well-being, compared to non-injured players (median 9; Q1–3 4), (*p* = 0.03). For current injuries, the binary regression analyses showed a significant association with total wellness score (*p* = 0.03; OR, 1.18 [95% CI, 1.01–1.38]), with a small effect size (Cohen’s d = 0.44 [95% CI, 0.03–0.85]). However, no significant differences were observed between the groups in the individual well-being categories of stress, sleep, or fatigue (*p* ≥ 0.09). Female players reported significantly higher perceived stress levels compared to male players (*p* = 0.01), with a moderate effect size (Cohen’s d = 0.57 [95% CI, 0.15–0.98], whereas no significant sex differences were observed in other measures of perceived well-being; sleep and fatigue (*p* ≥ 0.39) (Table 3).

## 4. Discussion

This study examined the injury profile, training exposure, risk factors, and perceived well-being of youth padel players. The principal findings indicated a relatively high prevalence of overuse injuries, a modest association between well-being indicators and injury status, and sex-specific differences in perceived stress levels. The overall injury prevalence of 0.53 injuries per player is consistent with some previous findings in comparable youth racket sports [31,32]. The prevalence of injury also aligns with findings from studies on adult padel players [33]. However, substantial differences in both prevalence and incidence of injury across racket sports [33,34], largely due to methodological variations, make direct comparisons difficult. Overuse injuries predominated, accounting for 89% (49/55) of all reported injuries, a trend consistent with findings in other youth sports characterized by repetitive loading [17]. This pattern reflects the demands of padel, which involves continuous play, frequent directional changes, and prolonged rallies. These findings are in accordance with previous studies on adult padel players, which also identified a high proportion of overuse injuries [8,9,10]. The knee emerged as the most commonly affected anatomical region (n = 12), consistent with evidence indicating that deceleration, lunging, and lateral movements, which are key components of racket sports, place considerable stress on the knee joint [35,36,37]. However, these specific movement patterns were not assessed in the present study. Although less frequent, injuries to the foot/ankle and lower leg (n = 5) were associated with the highest severity scores (median = 44), underscoring the importance of these regions for movement and stability in padel. Upper extremity injuries (shoulder, wrist, elbow), while less prevalent, remain relevant due to the overhead and swinging motions inherent to the sport. No statistically significant associations were found between injury prevalence and racket changes or outdoor play, despite previous studies indicating that changes in equipment or playing surfaces may contribute to injury risk [9,38]. This may be due to the relatively small sample size, which limits the feasibility of subgroup analyses. Although male players exhibited a slightly higher injury prevalence (0.56 vs. 0.47 injuries per player), the difference was not statistically significant, a finding consistent with previous research indicating minimal sex-based differences in youth racket sport injuries when adjusted for exposure [34].

Players reported a mean weekly training exposure of 10 h, a volume comparable to that of other youth racket sports. For example, high-performance youth tennis players typically train between 8 and 12 h per week [34]. No significant differences were found between sexes in terms of total training time, match exposure, or ITL, indicating equitable training demands. The mean ITL of approximately 6076 arbitrary units (AU) per week substantially exceeds values previously reported for junior tennis athletes [19]. Prior research has shown that acute ITL increases of >1750 AU per week, as well as weekly loads within the 3000–5000 AU range, are associated with elevated injury risk [39,40]. While adult athletes typically tolerate higher training loads and exhibit greater injury rates [40], the median RPE of 5 (on a 10-point scale) suggests that players perceived training as moderately intense and well-tolerated. Interestingly, female players were significantly older than male players, potentially reflecting sex-specific maturation patterns, as female athletes often reach peak performance at younger chronological ages [41]. Despite similar training volumes and loads, female players reported slightly higher levels of perceived stress, a finding consistent with literature indicating that adolescent females are more susceptible to psychological stress related to performance demands, social pressures, and physiological development [42,43]. No significant sex differences were observed in sleep quality or fatigue scores, suggesting comparable recovery profiles across groups.

An interesting finding was that injured players reported slightly higher total wellness scores (median 10) compared to non-injured players (median 9; *p* = 0.03), suggesting a potential link between injury status and overall well-being. Previous research has shown that higher perceived well-being is associated with a reduced risk of injury among female adolescent athletes [13,18,44]. One study found that a female athlete with a prior injury and low perceived well-being had a 48% likelihood of belonging to the ‘high injury’ group, compared to just 7% for a male athlete with no previous injury and high well-being [44]. These athletes were 15–16 years old and represented a diverse range of sports, including bandy, basketball, canoeing, curling, football, gymnastics, ice hockey, orienteering, sailing, skiing, swimming, tennis, and volleyball [44]. This result underscores the potential influence of psychological factors, such as perceived well-being, in relation to sex differences in injury vulnerability. It also reinforces the value of incorporating well-being assessments into training routines and risk management strategies. However, the effect size was small (Cohen’s d = 0.44) and no significant differences were observed in individual well-being components, namely stress, sleep, and fatigue, between groups in the present study. This underscores the multifactorial and complex nature of injury risk in youth athletes, where physical load, biomechanics, psychological stress, and external factors interact in dynamic ways [45].

Overall, these findings emphasize the importance of monitoring both objective and subjective measures of training load and well-being in youth padel players. Incorporating regular assessments of well-being into training programs may facilitate early identification of at-risk athletes and inform targeted injury prevention strategies. Future research should aim to elucidate causal relationships and develop validated screening tools for use in youth racket sports.

### Limitations and Future Directions

This study provides valuable insight into the training and injury patterns of youth padel players, but several limitations must be considered. First, the data are based on self-report, which may introduce response bias. Second, the cross-sectional design precludes causal inference regarding training load and injury risk. Third, although the Hooper Index has been widely employed as an instrument for assessing well-being in both adult [29,46,47] and youth athletes [48,49,50], its reliability has been examined exclusively in adult populations [29]. Moreover, this study is limited to Swedish padel players, which may affect the generalizability of the findings to players from other countries or cultural contexts. In addition, since this was an exploratory study that invited all licensed players aged 15–20 years, we did not control for the level of play. Different competitive levels may involve varying training loads, which could have influenced the results. Furthermore, male players are overrepresented in the present study (66%). However, this distribution is comparable to that of the overall cohort of Swedish padel players in the same age range, in which male players represent 73%. Future studies with larger cohorts are needed to determine whether there are sex-specific differences in injuries based on training hours, match hours, or RPE. Lastly, external factors such as biomechanics, coaching quality, and training surfaces were not fully explored. Future research should adopt longitudinal designs with objective load monitoring (e.g., GPS, heart rate, accelerometry), injury verification by healthcare professionals, and consider biomechanical and psychological screening. Interventions focusing on stress management and injury prevention (e.g., neuromuscular training) may also be warranted, especially for female athletes reporting elevated psychological stress.

## 5. Conclusions

Youth padel players are exposed to relatively high weekly training loads and show a notable prevalence of overuse injuries, especially in the lower extremities. No associations were found between injury risk and playing surface or recent racket changes. However, poorer perceived well-being was slightly associated with current injuries, suggesting a possible link between well-being and injury risk. Given that female players report slightly higher stress levels despite similar training volumes, it may be relevant for female coaches to actively engage in creating supportive environments that address the unique psychological demands faced by young women in padel. These findings suggest the importance of a more integrated approach to monitoring youth padel players, combining data on training load, well-being, and injuries. Coaches are encouraged to consider incorporating routine wellness assessments into training programs to better support healthy development and reduce injury risk.

## Figures and Tables

**Table 1:** Players’ age, self-reported weekly hours of training and games and internal training load presented with mean (SD) and rating of perceived exertion (RPE) presented with median together with q1–3 (Q).

	Women (n = 36)	Men (n = 68)	Total (n = 104)
	Mean (SD)	Mean (SD)	Mean (SD)
Age (y)	18 (1)	17 (1) *	17 (1)
Padel training (h)	6 (3)	7 (4)	6 (3)
Game (h)	3 (2)	4 (2)	3 (3)
Total training (h)	6 (4)	7 (5)	7 (5)
Internal training load (AU)	6047 (5724)	6092 (4456)	6076 (4904)
	**Median (Q)**	**Median (Q)**	**Median (Q)**
RPE training	5 (4–7)	5 (4–7)	5 (4–7)
RPE game	5 (4–7)	5 (5–7)	5 (4–7)

* = significant difference between groups.

**Table 2:** Location and number of overuse problems, including severity score, presented with median together with q1–3 (Q).

	Women (n = 9)	Men (n = 26)	Total (n = 35)	Severity Score
Injured Area	n	n	n	Median (Q)
Hip	2	3	5	37 (20.5)
Knee	3	9	12	43 (39)
Foot/ankle and lower leg	3	2	5	44 (62)
Lower back	2	8	10	34.5 (25.5)
Shoulder	1	3	4	29.5 (47.25)
Neck/upper back	1	1	2	18 (4)
Hand/wrist	3	4	7	30 (30)
Elbow	1	3	4	18 (35)
Total	16	33	49	

**Table 3:** Perceived well-being: stress, sleep quality, and fatigue during the past week and total wellness score presented with median together with quartiles (Q).

	Women (n = 36)	Men (n = 68)	Total (n = 104)
Stress	3 (2–4)	2 (1–4) *	3 (2–4)
Sleep quality	3 (2–4)	3 (2–4)	3 (2–4)
Fatigue	3 (2–4)	3 (2–4)	3 (2–4)
Total wellness score	9.5 (7–11)	9 (6–11)	9 (7–11)

* = significant difference between groups.

## Data Availability

The original contributions presented in the study are included in the article/Supplementary Material, further inquiries can be directed to the corresponding author/s.

## Supplementary Materials

The following supporting information can be downloaded at: https://www.mdpi.com/article/10.3390/sports13100356/s1.

## Abbreviations

The following abbreviations are used in this manuscript:

RPE	Rating of Perceived Exertion
ITL	Internal Training Load
AU	Arbitrary Units
