# Supplementary material for: Training Load, Injuries, and Well-Being in Youth Padel Players: A Cross-Sectional Study
Source: Sports (Basel). 2025 Oct 7;13(10):356. doi: 10.3390/sports13100356 (PMC12567576; doi:10.3390/sports13100356)
Supplement: Supplementary file 1 [file sports-13-00356-s001.zip › sports-3792576-supplementary.pdf]

Supplementary S1.

Padel-specific injury form, modified from Ryman Augustsson & Lundin (9) and Clarsen et al. (25).

Part 1

1. Have you suffered an **acute injury** during padel in the last 7 days (sudden event such as sprain, fall, strain)? (Yes/No).

2. (if yes) Do you know what type of acute injury?

   - No, don't know
   - Yes, ligament injury (hand)
   - Yes, ligament injury (shoulder)
   - Yes, ligament injury (knee, anterior cruciate ligament injury)
   - Yes, other ligament injury (knee)
   - Yes, ligament injury (foot)
   - Yes, fracture in foot, lower leg, or thigh bone
   - Yes, fracture in hand, arm, or shoulder
   - Yes, fracture in another location
   - Yes, meniscus injury (knee)
   - Yes, cartilage injury (knee)
   - Yes, muscle strain (calf)
   - Yes, muscle strain (front thigh)
   - Yes, muscle strain (back thigh)
   - Yes, muscle strain (arm or shoulder)
   - Yes, tendon rupture (Achilles tendon)
   - Yes, tendon rupture (shoulder, e.g., rotator cuff rupture)
   - Yes, tendon rupture (other)
   - Yes, facial injury
   - Yes, eye injury
   - Yes, head injury
   - Yes, concussion
   - Yes, other acute injury (not listed above)

3. Have you had any other problems, pain, or injury during the last 7 days? (Yes/No).

Part 2 (if 'Yes' is selected for Question 3)

**Question 1: Have you had any difficulties participating in normal padel training and/or competition due to x problem during the last 6 months?**
Full participation without x problems
Full participation, but with x problems
Reduced participation due to x problems
Cannot participate due to x problems

Question 2: To what extent have you reduced your training volume due to x problem during the last 6 months?

No reduction
To a minor extent
To a moderate extent
To a major extent
Cannot participate at all

Question 3: To what extent have x problem affected your padel performance during the last 6 months?

No effect
To a minor extent
To a moderate extent
To a major extent
Cannot participate at all

Question 4: To what extent have you experience x pain related to padel during the last 6 months?

No pain
Mild pain
Moderate pain
Severe pain

Question 5: To what extent have x problem affected your everyday activity during the last 6 months?

No effect
To a minor extent
To a moderate extent
To a major extent

X= hip, knee, foot/ankle/ lower leg, lower back, shoulder, neck/upper back (thoracic spine problems), hand/wrist/forearm and elbow problem.
